# Supplementary material for: Trypsin may be associated with duodenal eosinophils through the expression of PAR2 in early chronic pancreatitis and functional dyspepsia with pancreatic enzyme abnormalities
Source: PLoS One. 2022 Oct 20;17(10):e0275341. doi: 10.1371/journal.pone.0275341 (PMC9584419; doi:10.1371/journal.pone.0275341)
Supplement: S1 Checklist — (DOCX) [file pone.0275341.s001.docx]

# Author Formatting Checklist

Congratulations on your acceptance to *PLOS ONE*! To prevent article processing delays, we kindly ask that you use this checklist to help ensure that your submission files meet the journal’s formatting requirements.

[**References**](https://journals.plos.org/plosone/s/submission-guidelines#loc-references)

Is every item in the reference list cited in the main text? The reference list should only contain items cited in the main text. (References cited only in the Supporting Information should appear in a separate reference list within the supporting information.)

Are all reference citations denoted using the reference number in square brackets? Please do not use an author-year citation style.

Do all reference citations appear in the manuscript in ascending numerical order?

[**Tables**](https://journals.plos.org/plosone/s/tables)

Are all tables editable, cell-based objects? Tables in graphic format cannot be typeset. (Any graphic items must be submitted as figures or as supporting information.)

Are all tables cited in numeric order within the main text?

[**Figures**](https://journals.plos.org/plosone/s/figures)

Are your figures clear and legible, including the text? Do they follow PLOS figure requirements?

Does each figure match its corresponding caption and citation in the main text?

Are all figures cited in numeric order?

[**Supporting Information**](https://journals.plos.org/plosone/s/supporting-information)

Are your Supporting Information files clear of tracked changes? Supporting Information files will be published exactly as provided.

If your paper includes Appendices, are they included as Supporting Information files? Appendices must be within the SI and not the main manuscript text.

For more information about formatting requirements, please see *PLOS ONE’s* [submission guidelines](https://journals.plos.org/plosone/s/submission-guidelines), [formatting template](https://journals.plos.org/plosone/s/file?id=wjVg/PLOSOne_formatting_sample_main_body.pdf), and [figure guidelines](https://journals.plos.org/plosone/s/figures). If you have any questions, please contact us at plosone@plos.org.
