# Supplementary figures and images for: Trypsin may be associated with duodenal eosinophils through the expression of PAR2 in early chronic pancreatitis and functional dyspepsia with pancreatic enzyme abnormalities
Source: PLoS One. 2022 Oct 20;17(10):e0275341. doi: 10.1371/journal.pone.0275341 (PMC9584419; doi:10.1371/journal.pone.0275341)

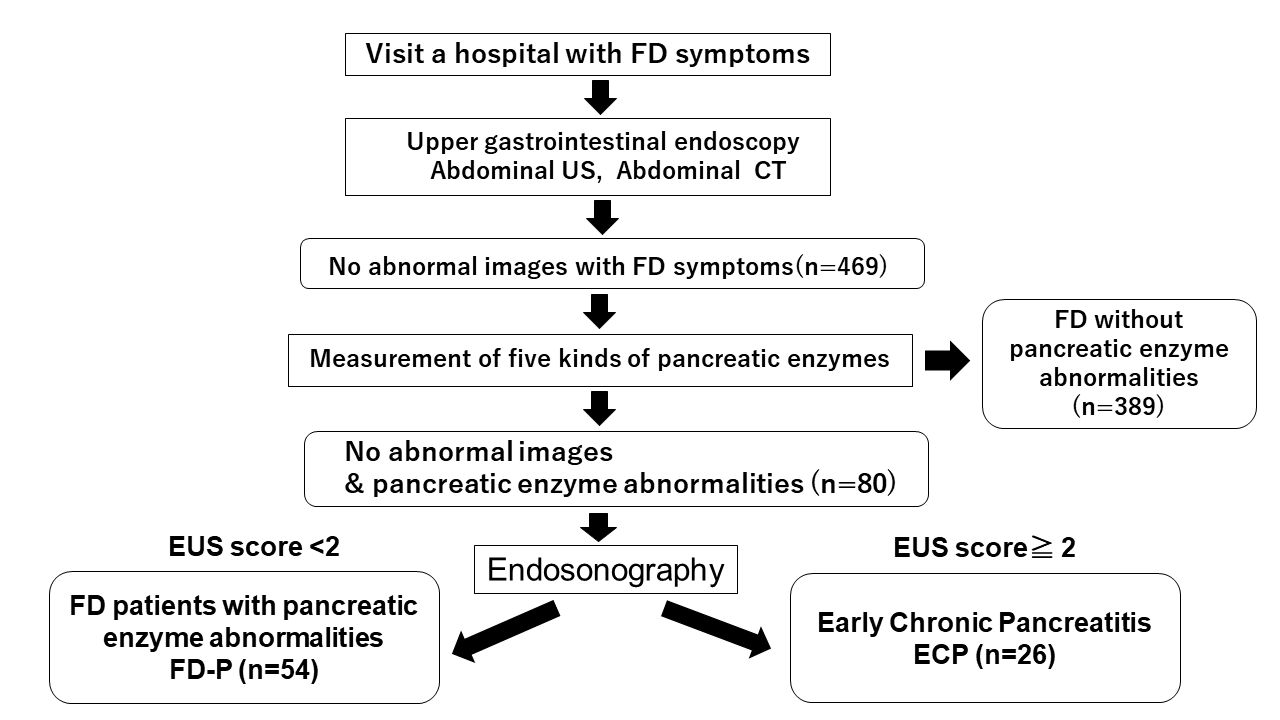

Supplement: S1 Fig — (TIF) [file pone.0275341.s002.tif]

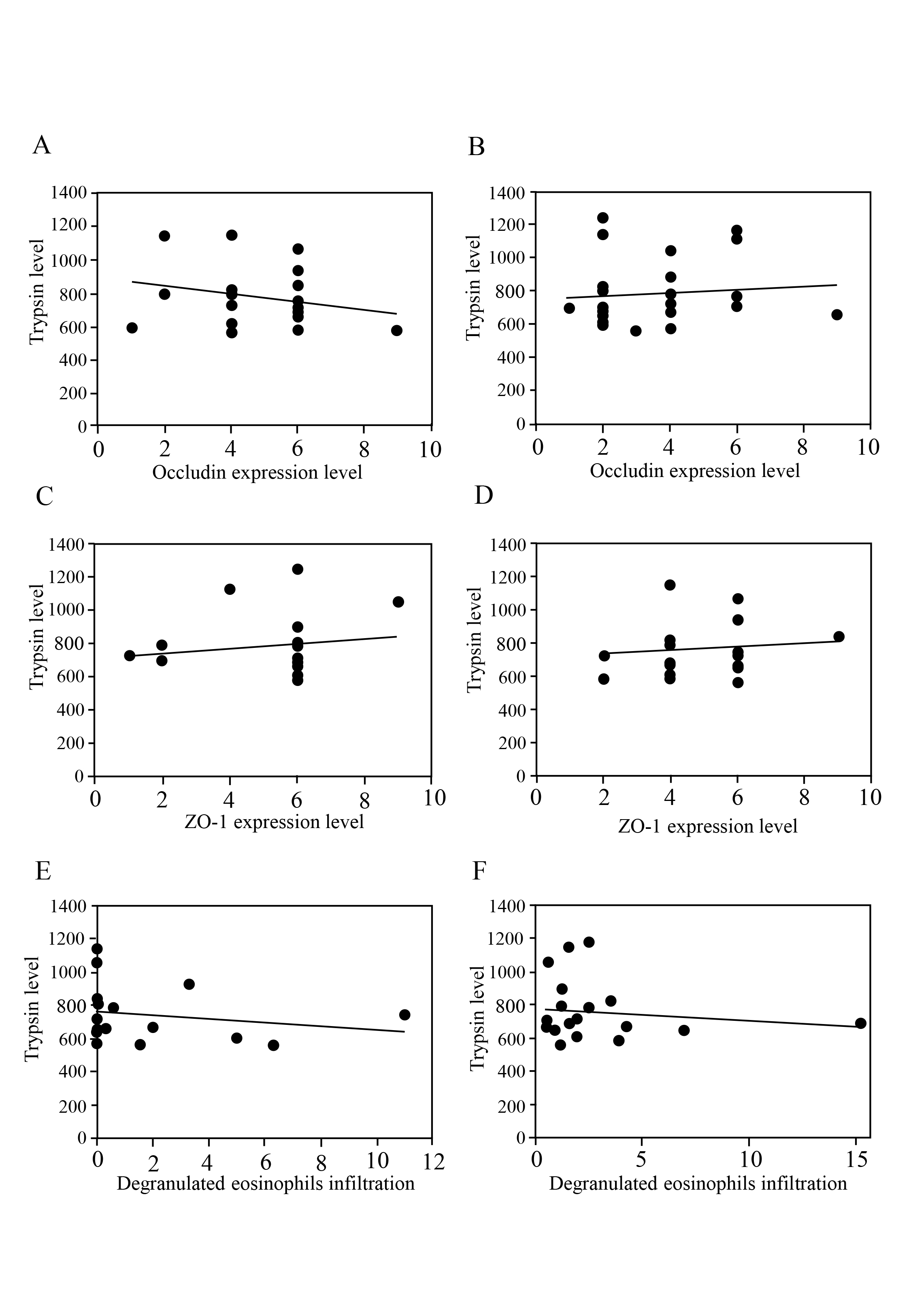

Supplement: S2 Fig — There was no significant relationship between elevated trypsin levels and occlusion or ZO-1 expression levels in ECP and FD-P patients (S2A, S2B, S2C, and S2D Fig). In addition, elevated trypsin levels were not associated with degranulated eosinophils infiltration in ECP and FD-P patients (S2E and S2F Fig). These groups were compared using a correlation coefficient. (TIF) [file pone.0275341.s003.tif]
